# Does duration of untreated illness impact long-term outcome in obsessive-compulsive disorder?

**DOI:** 10.1192/j.eurpsy.2024.733

**Published:** 2024-08-27

**Authors:** S. Cipolla, P. Catapano, S. Pascolo, M. Luciano, G. Sampogna, F. Perris, V. Giallonardo, V. Del Vecchio, M. Fabrazzo, A. Fiorillo, F. Catapano

**Affiliations:** ^1^Psychiatry, University of Campania “L. Vanvitelli”, Naples, Italy

## Abstract

**Introduction:**

The time period between the onset of a mental disorder and its first adequate treatment (duration of untreated illness - DUI) influence long-term prognosis and outcome in patients with severe mental disorders. The relationship between DUI and outcome was originally found in people affected by schizophrenia spectrum disorders, however in patients with Obsessive-Compulsive Disorder (OCD) DUI is significantly longer compared with that of patients with other severe mental disorders, such as schizophrenia and bipolar disorder.

**Objectives:**

Aims of the present study is to assess the impact of DUI on long-term outcomes in OCD patients across published studies.

**Methods:**

A systematic review was carried out by selecting relevant articles on the topic present in three common on-line databases, such as PubMed, APA PsycInfo, and Scopus, up to June 2023.

**Results:**

Among included studies, DUI ranged from 7,0±8,5 to 20,9±11,2 years. Patients reporting a longer DUI have a poor long-term outcome, in terms of greater symptom severity and lower level of treatment response, whether pharmacological treatment or psychotherapy or a combination of these two. This is particularly true when the onset of the disease is insidious and subthreshold. However, there are severe early-onset forms of OCD in which the request for help is anticipated due to the severity of the symptoms, the DUI is shorter, but the prognosis is still negative.

**Conclusions:**

The present review confirms that longer DUI has a negative impact on the long-term outcome of patients with OCD. Furthermore, it is reasonable to hypothesize that cultural factors, such as the perception of the disease and the ability to access treatment, may result in a prolongation of the DUI. All these elements cannot be evaluated in our review due to the paucity of studies on the topic. Future studies could be useful to better understand the causes of a longer DUI, to guide and to promote the dissemination of early interventions with a specific focus on OCD symptoms.

**Disclosure of Interest:**

None Declared

